# Therapy-Induced Tumor Cell Death: Friend or Foe of Immunotherapy?

**DOI:** 10.3389/fonc.2021.678562

**Published:** 2021-06-01

**Authors:** Thijs A. van Schaik, Kok-Siong Chen, Khalid Shah

**Affiliations:** ^1^ Center for Stem Cell Therapeutics and Imaging (CSTI), Harvard Medical School, Boston, MA, United States; ^2^ Department of Neurosurgery, Brigham and Women’s Hospital, Harvard Medical School, Boston, MA, United States; ^3^ Harvard Stem Cell Institute, Harvard University, Cambridge, MA, United States

**Keywords:** immunotherapy, tumor cell death, tolerogenic, immunogenic, tumor microenvironment (TME), damage associated molecular pattern (DAMP), caspase-dependent apoptosis, therapy-induced senescence

## Abstract

Combinatory treatments using surgery, radiotherapy and/or chemotherapy together with immunotherapy have shown encouraging results for specific subsets of tumors, but a significant proportion of tumors remains unsusceptible. Some of these inconsistencies are thought to be the consequence of an immunosuppressive tumor microenvironment (TME) caused by therapy-induced tumor cell death (TCD). An increased understanding of the molecular mechanisms governing TCD has provided valuable insights in specific signaling cascades activated by treatment and the subsequent effects on the TME. Depending on the treatment variables of conventional chemo-, radio- and immunotherapy and the genetic composition of the tumor cells, particular cell death pathways are activated. Consequently, TCD can either have tolerogenic or immunogenic effects on the local environment and thereby affect the post-treatment anti-tumor response of immune cells. Thus, identification of these events can provide new rationales to increase the efficacy of conventional therapies combined with immunotherapies. In this review, we sought to provide an overview of the molecular mechanisms initiated by conventional therapies and the impact of treatment-induced TCD on the TME. We also provide some perspectives on how we can circumvent tolerogenic effects by adequate treatment selection and manipulation of key signaling cascades.

## Introduction

In the last decade, incredible steps forward have been made in the advances of cancer treatment. Although combinatory treatments using surgery, radiotherapy and/or chemotherapy together with immunotherapy have shown encouraging results for specific subsets of tumors, a significant proportion of tumors remains unsusceptible or becomes resistant to therapy. Therefore, more efforts are required to understand the underlying mechanisms that influence the outcome of these treatments. One possible explanation for these inconsistencies could be the dynamics of the tumor microenvironment (TME) post treatment (1, 2). The TME has been shown to play a pivotal role in cancer development, progression, immunity and treatment (3). These events are governed by tumor cells and a variety of non-malignant cells such as immune cells, fibroblasts and vasculature cells, which are hijacked by the tumor cells. Consequently, the non-malignant cells in the TME display altered phenotypes (4) and cause an immunosuppressive environment (5, 6). The balance of physical and chemical interactions regulating hijacking and immunosuppression is tightly regulated (3), but this equilibrium shifts when major physiological changes occur.

One process that appears to be a major contributor to these biochemical changes is treatment-induced tumor cell death (TCD), as dying cancer cells have been shown to release a wide variety of regulatory signals that affect both tumor and stromal cells (7). For example, a combination of two chemotherapeutic agents increases immune suppression in the TME as a result of secreted factors from dying tumor cells. These molecules stimulate regulatory T (T_reg_) cell proliferation and increase the number of immune-suppressive dendritic cells (DCs) (8). In this review, we discuss the effects of different types of TCD induced by conventional therapies on the immune cells in the TME. Additionally, we provide perspectives on how this knowledge can help in the optimization of conventional therapies and the design of new combinatory treatments for cancer.

**Figure 1:**
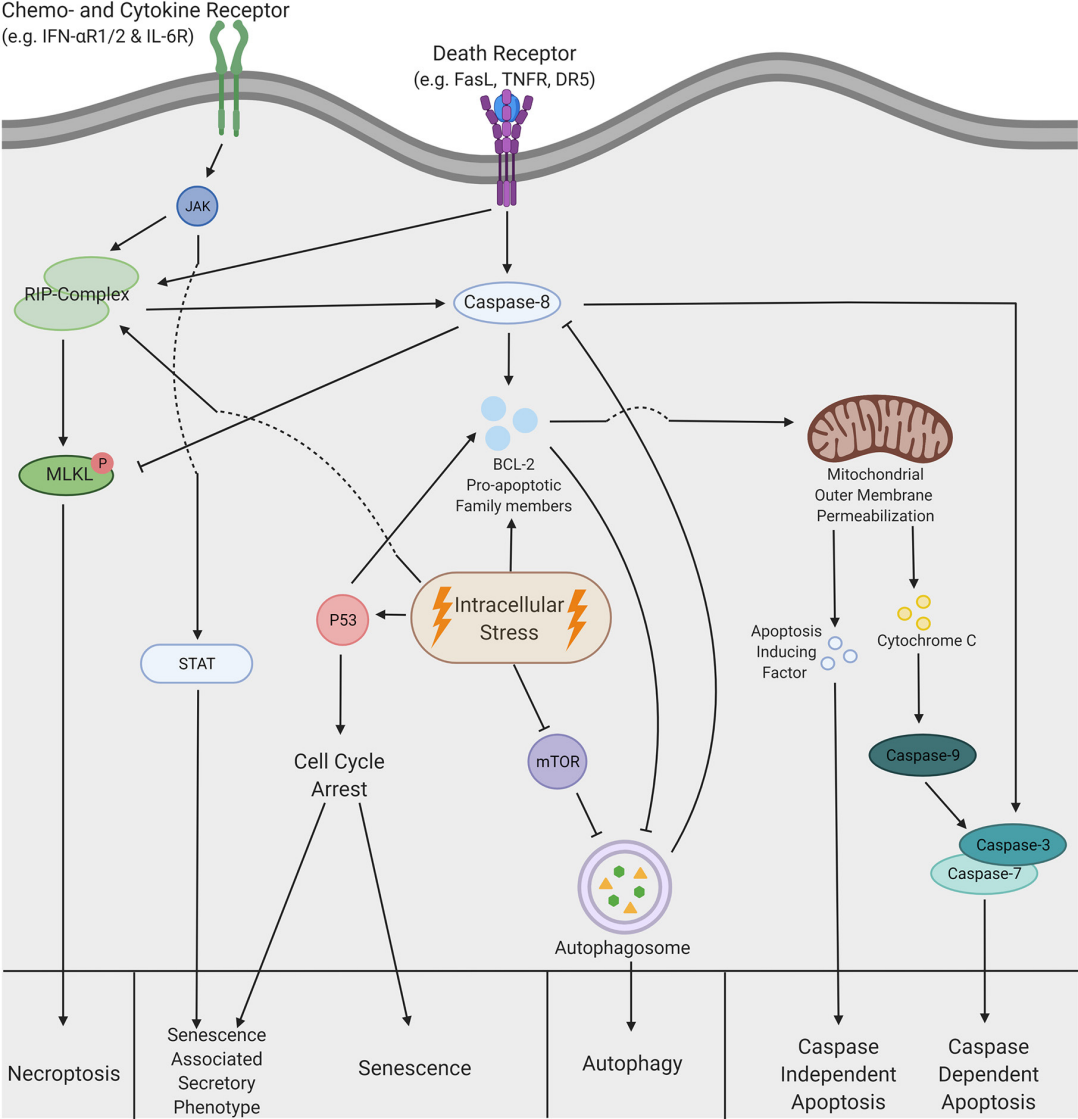
A schematic overview of the complex pathways regulating cell death. Necroptosis can be triggered by both extrinsic and intrinsic stimuli. The extrinsic pathway can be activated by chemokine, cytokine and death receptors, which leads to the activation of receptor interacting protein (RIP)-kinases. Similar activation is also achieved *via* intracellular stress. Subsequently, activation of the RIP-complex leads to phosphorylation of mixed lineage kinase domain like pseudokinases (MLKL), which is inhibited by caspase-8 under normal circumstances. MLKL phosphorylation induces a translocation to the cell membrane, which leads to permeabilization of the cell and consequently necroptotic cells death. Senescence is predominantly induced *via* a cell cycle arrest initiated by p53, but this process can also lead to the senescence associated secretory phenotype (SASP). Moreover, SASP can be induced *via* extrinsic stimuli that activate the Janus kinase- signal transducer and activator of transcription (JAK-STAT) pathway. Intracellular stress can inhibit the mammalian target of rapamycin (mTOR) pathway, which allows formation of the autophagosome. This formation can be inhibited by pro-apoptotic members of the BCL-2 family but is also able to inhibit caspase cleavage and thereby displays the crosstalk between autophagy and apoptosis. Eventually, after formation of the autophagosome, autophagic cell death is induced. Additionally, apoptosis can be induced by both intrinsic and extrinsic stimuli. Extrinsic stimuli allow cleavage of caspase-8, which can either induce cleavage of the caspase3/7 complex or activate members of the pro-apoptotic BCL-2 family. Activation of caspase3/7 results in caspase dependent apoptosis. However, the BCL-2 proteins can also be activated by intrinsic stimuli or intracellular stress and lead to the mitochondrial outer membrane permeabilization (MOMP), which in turn releases both cytochrome C and apoptosis inducing factors (AIF). Cytochrome C interacts with caspase-9 and subsequently induces caspase-dependent apoptosis *via* the activation of caspase 3/7. In contrast, AIF facilitates caspase-independent apoptosis by initiation of chromatin condensation and DNA fragmentation. This figure was created by authors using Biorender tools (biorender.com).

Based on the morphological changes and biochemical features, there are five major types of cell death (9, 10). Regulating pathways of these distinct forms are complexly intertwined and are summarized in **Box 1** and **Figure 1**. The TME is influenced by each type of cell death differently and depending on the pathway of activation, these effects range from tolerogenic to immunogenic properties, which are considered as ‘silent’ or ‘loud’ respectively. The latter attracts immune cells *via* the secretion of damage associated molecular patterns (DAMPs) and allows immune cells to provoke an anti-tumor response, which is clinically beneficial (29). Tolerogenic stimuli facilitate the eradication of dead cells without provoking an immune response and are often accompanied by the release of immunosuppressive chemo- and cytokines (30). The generalized effects of the five types of cell death on the TME and their subsequent influence on immune cells in this environment are summarized in **Table 1**.

Box 1 **Overview Molecular Mechanisms Regulating Cell Death**

Necroptosis

Necroptosis can be initiated by both intrinsic and extrinsic stimuli that activate receptor-interacting protein (RIP)-kinases (11, 12). These can act together or interact with the caspase pathways, but necroptosis is only activated when RIP-kinases act independently of caspase-8 (13). Subsequently, RIP-kinases activate mixed lineage kinase domain like pseudokinases (MLKL), which form protein-complex, resulting in permeabilization of the cell membrane (14, 15).

Senescence

Cell death can also occur *via* senescence, which is often activated by p53 and cyclin dependent kinase, and results in a permanent cell-cycle arrest (16). This signaling cascade was found to induce both senescence and senescence associated secretory phenotype (SASP). Furthermore, activation of the Janus kinase-signal transducer and activator of transcription (JAK-STAT) pathway results in the transformation to the SASP in both cancer cells and healthy cells (17, 18).

Autophagy

Autophagy is tightly regulated by mammalian target of rapamycin (mTOR), members of the pro-apoptotic BCL-2 family (19, 20) and caspase-8 (21) which is essential for homeostasis in healthy cells (22). After autophagy is initiated, an autophagosome is formed where intracellular structures are degraded (23). Consistent activation of the autophagy machinery eventually leads to autophagic mediated cell death.

Apoptosis

Apoptosis is induced *via* two distinct pathways. The caspase-dependent pathway is either activated *via* ligand receptor binding of a death receptor complex (24) or though high levels of intracellular stress. Death receptors cleave caspase-8, which can interact with other members of the caspase cascade or initiate apoptosis *via* the BCL-2 family. Activation of the proapoptotic BCL-2 protein family members also occurs through intracellular stress. These proteins induce mitochondrial outer membrane permeabilization (MOMP) (25), which leads to the secretion of both apoptosis inducing factors (AIFs) and cytochrome C. The latter activates the caspase-cascade, but AIFs induce caspase-independent apoptosis.

Mitotic Catastrophe

Aberrant mitosis during the M-phase can lead to a specific mode of cell death termed mitotic catastrophe. This process is frequently caused by defects in the cell-cycle (26). Once a certain threshold of lethal signals is reached, the cell will die *via* pathways that regulate senescence, necroptosis and apoptosis (27), and relies on caspase-dependent and independent pathways (28).

**Table 1:** Overview of effects of tumor cell death to the tumor microenvironment.

Type of Cell Death	Subtype	General Effect on TME	Molecular Effects	Consequences in TME	Reference
Apoptosis	Caspase-Dependent	Tolerogenic	Tolerogenic ‘find me’-signals & Tolerogenic ‘eat me’-signals	Silent phagocytosis by macrophages	(31, 32)
Apoptosis	Caspase-Independent	Immunogenic	HMGB1 release and Extracellular CRT	Clearance by DCs and macrophages and increased cross-presentation	(30)
Necroptosis		Immunogenic	ATP and HGMB1 release	DC and T cell maturation	(33)
Autophagy		Both	Degradation of CRT and HMGB1 & ATP and HMGB1 release	Increase of T cell activation	(34, 35)
Senescence	SASP	Tolerogenic	Secretion of IL-6, IL-1, IGF, MMPs and CXCL8	Inhibition of the anti-tumoral T cell and NK cell response and attraction of MDSCs	(36)
Mitotic Catastrophe		Unknown	Unknown	Unknown	–

TME, Tumor Microenvironment; SASP, Senescence Associated Secretory Phenotype; HGMB1, High Mobility Group Box 1 protein; CRT, Extracellular Calreticulin; ATP, Adenosine triphosphate.

## Consequences of Therapy-Induced TCD

Conventional treatments exploit the molecular mechanisms of cell death and therefore have a type-specific effect on the TME. In this section, we focus on the immunogenic or tolerogenic effects on the TME elicited by chemotherapy, radiotherapy and immunotherapy. Comprehension of the molecular mechanisms governing therapy-induced TCD and their consequences can lead to a better understanding of treatment effects, and is therefore crucial for the design of effective anti-cancer therapies.

### Chemotherapy

Chemotherapy is the most widely used cancer treatment and induces all five types of TCD depending on the activated molecular mechanisms (37). Most chemotherapeutic agents act on the genomic content of tumor cells to inhibit proliferation and thereby induce cytotoxicity (38). After interfering with processes such as DNA synthesis, microtubule formation and cell division, numerous TCD cascades can be activated. Chemotherapeutic agents induce apoptosis either *via* the caspase-dependent and independent pathway or both simultaneously (39–41). For example, caspase-dependent apoptosis has been observed after treatment of lymphoma cells with paclitaxel (42), whereas the DNA-damaging agent cisplatin and protein synthesis inhibitor doxycycline activate both the caspase-dependent and -independent pathways (43, 44).

Caspase-independent apoptosis stimulates the upregulation of immune-activating molecules or DAMPs, such as high mobility group box 1 (HMGB1), heat shock proteins (HSPs) and extracellular calreticulin (CRT) (45, 46). These stimuli initiate an immune response *via* DCs antigen presentation. Mutations in the DAMP-recognizing receptors on DCs were associated with earlier relapse after chemotherapy in breast cancer patients (47). This emphasizes the fact that an effective immune response is necessary for a successful treatment outcome. However, the majority of conventional chemotherapeutic agents act on the caspase-dependent pathways, which results in silent phagocytosis by anti-inflammatory phagocytes that are recruited by tolerogenic ‘find-me’ signals (31) and facilitated by immune-suppressive ‘eat-me’ signals (32). The induction of tolerogenic TCD and thus an improper immune response could therefore be an explanation for the lower progression-free survival in patients treated with caspase-dependent apoptosis inducing agents (48–50).

Autophagy can be initiated by various chemotherapeutic agents such as temozolomide and oxaliplatin (51, 52), but the contribution of autophagy to the therapeutic effects of chemotherapy remains unclear. For example, defects in the autophagic pathways impede the release of HMGB1, as well as the secretion of tolerogenic cytokines such as IL-1β and IL-6 (34, 53, 54). Moreover, an increase in ATP and HMGB1 has been found after chemotherapy-induced autophagic TCD in immune-competent mice (55). These results suggest that induction of autophagic TCD by chemotherapeutic agents can cause an immunogenic transformation of the TME, but also emphasize the need for a better understanding of the autophagic machinery and the interaction with other forms of cell death. A frequently suggested justification for the discrepancies is that some tumors rely on autophagy more heavily in terms of metabolism due to genetic alteration or nutrient deprivation in the TME (56), which could affect the degradation and secretion of immunomodulatory signals.

Conventional chemotherapies such as 5-FU and cisplatin also induce necroptosis in tumor cells (57, 58). For instance, disruption of the necroptotic pathway reduces chemotherapy-induced TCD and significantly decreases the release of DAMPs in mice (59, 60). The DAMPs released after necroptosis mainly consist of HMGB1 and ATP, which promote DC and T cell infiltration and maturation *via* antigen cross-presentation (13). This leads to a potent anti-tumor response and efficient long-term immunity (33, 61). Unfortunately, various cancer types have displayed silencing of the key regulators in the necroptotic pathway by DNA hypermethylation, which diminishes the chemosensitivity and the immunogenic response, and results in a lower overall survival in patients with various forms of cancer (62, 63). Together, necroptosis has been found to induce an immunogenic effect after chemotherapy, but co-occurrence with apoptosis post-chemotherapies minimized this immunostimulatory effect.

Another substantial consequence of chemotherapy is senescence, which is induced by DNA-damage due to cellular stress (64). Although senescence can contribute to the efficacy of chemotherapy (65), the transformation of tumor cells into the senescence associated secretory phenotype (SASP) negatively affects the clinical outcome (66). This transformation is mainly caused by DNA damage-induced senescence and activated *via* both p53-dependent and -independent pathways (67). Relative to other senescent tumor cells, SASP tumor cells have a distinct secretome including interleukins, inflammatory cytokines and growth factors such as IL-6, IL-1, IGF, MMPs and CXCL-8 (68, 69). These molecules promote tumor growth (70) and have predominantly tolerogenic effects, such as the inhibition of the anti-tumoral T cell response (36) and natural killer (NK) cell (71), as well as the attraction of myeloid derived suppressor cells (MDSCs) (72). For instance, a strong senescent response has been observed in breast cancer patients after docetaxel and doxorubicin treatment, which is caused by an increase in MDSC infiltration and a decrease in T cell activation and infiltration (66, 73). This data suggests that DNA-damaging chemotherapeutics induce senescence and the SASP simultaneously in tumor cells, which results in a tolerogenic effect on the TME (74).

In summary, chemotherapy predominantly induces caspase-dependent apoptosis complemented with caspase-independent apoptosis, autophagy, senescence and/or necroptosis and can therefore lead to a wide range of effects on the TME. This intimate relationship is further emphasized by the findings that different forms of cell death occur in a time-dependent manner (75). For example, temozolomide used in glioblastoma treatment induces autophagy, senescence and apoptosis in sequential order (76), providing a possible framework for time-dependent administration of adjuvant therapy. Taken together, these studies accentuate the complexity of these intertwined pathways and demonstrate that the treatment efficacy relies on the type of chemotherapy, the induced signaling cascades and consequently the type of cell death. Moreover, in-depth knowledge of the effects of chemotherapy on the TME facilitates a decent fundament to design new combinatory strategies for chemoimmunotherapy.

### Radiotherapy

Treatment with ionizing radiation led to the discovery of the apoptotic pathway and was first thought to predominantly rely on the intrinsic apoptotic pathway (77). These highly energetic radio waves induce double-stranded breaks in the DNA and consequently activate the caspase-cascade of apoptosis (78). However, recent studies have shown that cellular stress induced by radiation can lead to activation of various types of TCD (79). Various parameters are thought to influence the activation of TCD pathways such as dose, number of fractions and type of radiation. For example, distinct forms of ionizing radiation such as UV-B and UV-C radiation induce caspase-independent cell death which partially contributes to the cellular susceptibility to radiation-induced TCD (80–82). These results demonstrate the variability induced by the type of radiation. However, direct associations between apoptosis, type of radiotherapy, DAMP release and the subsequent immune response are unexplored.

Although apoptosis is the most commonly examined form of cell death after radiotherapy, high levels of radiation have been shown to induce a substantial amount of necroptosis and subsequently HMGB1 release, which was not observed in a low dose treatment (83, 84). Furthermore, knock-down of the RIP-kinase pathway results in a decrease in extracellular HMGB1 and expression levels of RIP-kinases significantly correlates with progression-free survival in patients with non-small cell lung cancer after high dose radiotherapy (85). This demonstrates the pivotal role of necroptosis in effective radiotherapy and suggests an important contribution of released immunogenic factors, but very little data is available regarding its direct effects on the immune system.

An association between radiation and autophagy has also been demonstrated, but opposing outcomes in terms of clinical efficacy have been found due to the discrepancy between cytoprotecting and cytotoxic autophagy (86). For example, autophagy is a radioprotective mechanism as the inhibition of the machinery sensitized radioresistant cell lines for low radiation doses (87). However, superficial amounts of radiation induce cytotoxic autophagy (88), which is linked to the release of DAMPs in a dose-dependent manner. High doses of radiation increase the induction of cytotoxic autophagy and DAMP release, and thereby transform the TME towards a more immunogenic environment (89). These findings display that only high levels of radiation can exceed the cytoprotective effects of autophagy, which consequently activates immunogenic cell death. Yet, most studies have been performed *in vitro* and therefore, more clinical or *in vivo* evidence is necessary to verify these findings.

Radiation also induces mitotic catastrophe if cells have malfunctioning cell cycle checkpoints or DNA damage response (90). The consequences of mitotic catastrophe TCD induced by radiation are similar to either apoptosis or necroptosis depending on the pathway of activation, but specific effects of mitotic catastrophe on the TME remain understudied.

Another understudied type of TCD induced by radiation is senescence since subsequent effects on the TME remain unknown (91). However, high levels of radiation induce SASP in malignant cells, which leads to increased secretion of tolerogenic chemo- and cytokines (92). Furthermore, the amount of SASP cells and consequently levels of SASP-associated cytokines is associated with genetic deficiencies in regular senescent pathways such as p53 and PTEN (93, 94). This demonstrates the strong molecular interaction between regular senescence and SASP and stresses the need for tumor-specific treatments. In general, high levels of radiation induce SASP; but no direct correlations in mouse models or patients have hitherto been found between radiotherapy, these secreted factors and the recruitment of suppressive immune cells.

In conclusion, radiotherapy can induce all forms of TCD. The immune responses of radiation induced TCD in TME are impacted by the types and doses of radiotherapy, as well as the genetic alterations in tumor cells. Doses of highly energetic forms of radiation are linked to more immunogenic forms of TCD such as caspase-independent apoptosis, necroptosis and cytotoxic autophagy. Currently, stereotactic body radiation therapy (SBRT) is of high clinical interest due to the possibility to deliver extremely precise high doses of radiation. The hypothesis is that SBRT delivers considerably higher biological effective doses of radiation, which induces more immunogenic TCD and has been shown to work synergistically with PD-1 checkpoint inhibition (95, 96). Ongoing studies have yet to determine the optimal radiation and fractionation dose, but the abovementioned studies demonstrate the possibilities for improvement of the conventional way of treatment.

Although a wide variety of studies have been published in the field of radiotherapy and tried to address the complexity of radiotherapy-induced cell death; direct interactions, underlying mechanisms of TCD and the interplay with the immune system remain obscure. A deeper understanding of the molecular mechanisms involved in radiotherapy is necessary to develop new radioimmunotherapy strategies.

### Immunotherapy

The most common immunotherapies are checkpoint inhibitors and chimeric antigen receptor (CAR) T cells, which both rely on cytotoxic T cells to induce TCD (97, 98). Three major mechanisms are involved in cell death induced by cytotoxic T cells, namely FasL-dependent, Granzyme-mediated and chemokine-dependent cell lysis (99).

Activation of the FasL-pathway results in caspase cleavage and thus leads to tolerogenic apoptosis. However, if the caspase pathway is defective in tumor cells, necroptosis will be activated (100). For granzyme-mediated cell death, both the caspase-dependent and independent apoptotic pathway are involved depending on the type of granzyme secreted (101, 102). Cytotoxic T cells express a wide variety of granzymes and thus initiate both immunogenic and tolerogenic TCD simultaneously. Lastly, the cytokine-dependent lysis of cytotoxic T cells is primarily triggered by TNF-a, which normally activates the caspase-cascade (103). Similar to previously mentioned FasL-dependent necroptosis, TNF-a dependent necroptosis has been observed in tumor cells with deficiencies in the caspase pathway (104).

Although the immediate effects of T cell mediated TCD are known, implications of induced cytotoxicity on the TME remain understudied. More insights into the consequences of T cell-mediated TCD could result in new strategies to enhance immunotherapy and therefore increase the efficacy of this treatment in solid tumors. In contrast, CAR T cells are found to transform the TME after encountering their target epitopes. For example, activated CAR T cells secrete immunomodulatory cytokines such as GM-CSF and IFN-y (105). This results in the transformation of suppressive M2-macrophages to activated M1-macrophages and therefore a more immunogenic environment, which consequently increases macrophage mediated killing and recruitment of host T cells. Thus, tolerogenic forms of apoptosis exploited by T cells might contribute to the immunosuppressive TME, but T cells are also found to directly modulate this environment. Therefore, more evidence is needed to explain the disappointing results in the treatment of solid tumors with immunotherapy (106).

Recently, another form of immunotherapy has been approved by the FDA, namely an oncolytic virus targeting melanoma cells (107). These viruses are capable of replicate and destroy tumor cells selectively and thus spare healthy cells. Furthermore, various forms of immunogenic TCD are observed after oncolysis depending on the type of virus used.

For example, most adenoviruses (AVs) induce autophagic and necroptotic TCD (108, 109), and are found to induce a T cell-mediated anti-tumor response through the release of DAMPs such as ATP and HGMB1 (110, 111). Moreover, herpes simplex virus (HSV)-mediated cytotoxicity relies on autophagy and both caspase-dependent and -independent apoptosis, but inhibits necroptotic TCD (112–114). Various DAMPs are released after induction of these pathways such as HSPs, HMGB1 and CRT (115) and infection of tumor cells has been shown to improve the T cell-mediated anti-tumor immune response in tumor-bearing mice (116). Evidence for this immune response has also been found in HSV-treated patients with various solid tumors (117). Thus, oncolytic viruses initiate immunogenic TCD and display the potential to provoke a potent anti-tumor immune reaction.

Additionally, lysis of virally infected cells adds the advantage of released viral particles or pathogen associated molecular patterns (PAMPs) (118), which are also extremely immunogenic. On the other hand, PAMPs also induce viral clearance and emphasize the importance of adequate viral delivery to the tumor bulk. In conclusion, both the DAMP and PAMP release facilitate an effective immune reaction, which can be exploited to increase the effectiveness of anti-cancer treatment with for example the combination of adjuvant immunotherapy. Furthermore, the activation of multiple cell death pathways after infection again highlights a close relationship between apoptosis, autophagy and necroptosis, which was also found to be exploited by wild type viruses to increase replication (119). This property is often used to increase the effectiveness of treatments by upregulation of certain genes but should be considered carefully to ensure the maximum release of DAMPs *via* immunogenic forms of cell death.

In essence, chemotherapy, radiotherapy and immunotherapy are capable of inducing both tolerogenic and immunogenic cell death. These effects depend on the treatment type and the genetic alterations in the tumor cells. Henceforth, genetic and treatment variables should be taken into account when designing novel treatment strategies, especially when these standard therapies are combined with immunotherapy.

## Strategies to Avoid Tolerogenic TCD

Multiple studies have demonstrated the beneficial effects of immunogenic forms of cell death, caused by the release of DAMPs in the TME and the subsequent activation of immune cells. This provokes an immune reaction against tumor cells, which leads to long-lasting anti-tumor immunity and a decreased risk of tumor recurrence or metastasis (120). In contrast, tolerogenic TCD increases pro-tumor effects by inhibiting the anti-tumor immune response (36), which leads to a lower progression-free and overall clinical survival in cancer patients (121). Therefore, immunological TCD *via* necroptosis and caspase-independent apoptosis is more desirable than tolerogenic TCD *via* caspase-dependent apoptosis. Various strategies can be used to increase the immunogenic effects of TCD and establish reliable treatments. Here, we will discuss four strategies: avoiding therapies that induce tolerogenic effects, converting tolerogenic TCD to immunogenic TCD, boosting immunogenetic effects and regulation of the tolerogenic stimuli. Some of these strategies have already been exploited in the clinic, but most are demonstrated in pre-clinical models and are currently under clinical investigation.

### Avoiding Tolerogenic Therapies

As described above, most chemotherapies induce TCD *via* the tolerogenic caspase-dependent pathway and should be avoided. Given that some drugs could induce different forms of TCD, Wang and colleagues (122) summarized multiple chemotherapeutics that provoked an immune response *via* the upregulation of DAMPs. These drugs are found to act *via* various cytotoxic mechanisms, such as alkylating agents, antimetabolites and cytotoxic antibiotics, and could be exploited in a wide range of cancer types.

**Figure 2:**
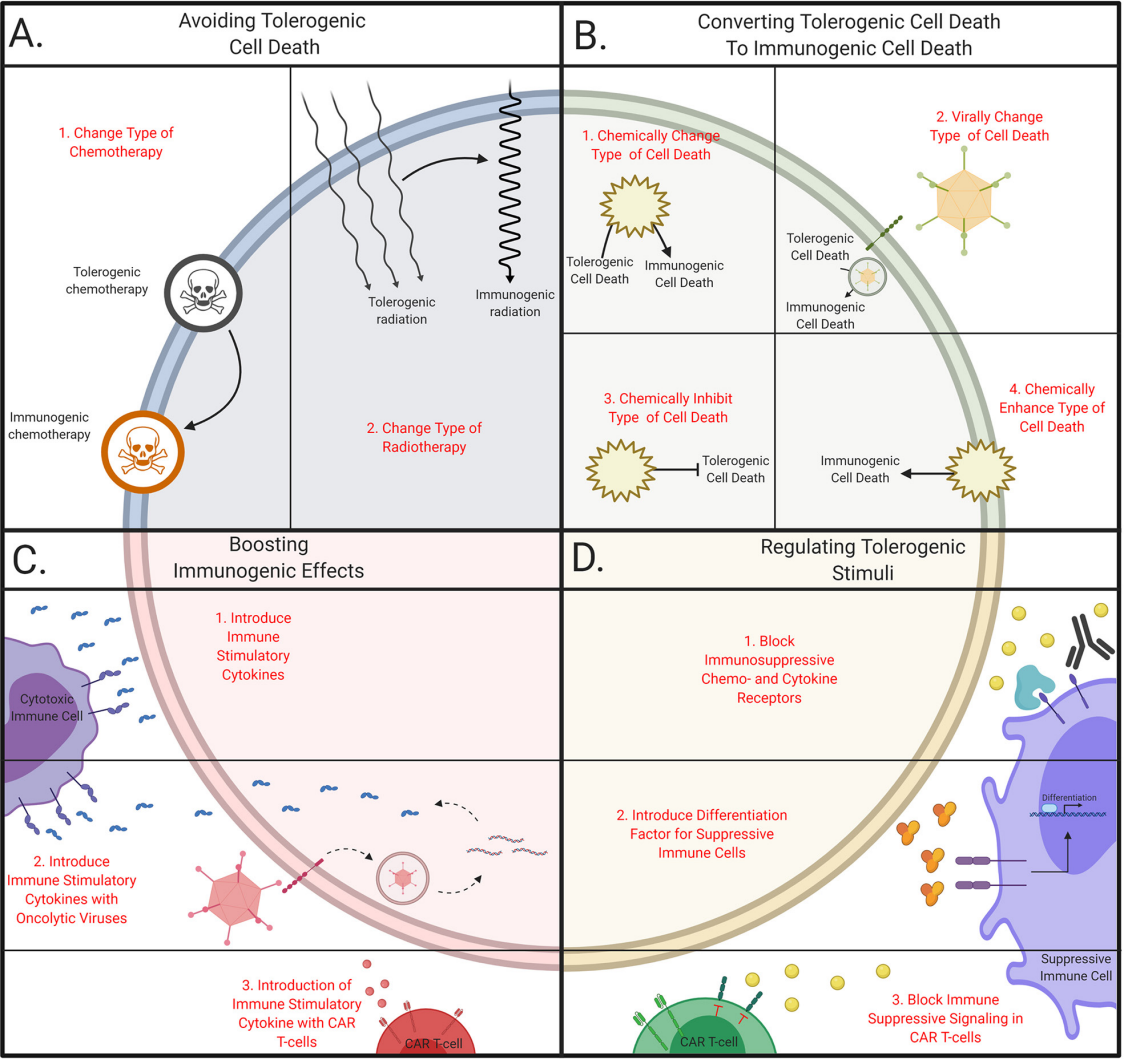
Schematic overview of tailoring therapeutic strategies to avoid tolerogenic cell death. **(A)** Avoiding tolerogenic cell death. Immunogenic cell death can be induced by (1) changing chemotherapeutic agents or (2) changing dose and regimes in radiotherapy. **(B)** Converting tolerogenic cell death to immunogenic cell death. Immunogenic cell death can be initiate by (1) chemically or (2) virally changing the cell death pathway, (3) chemically inhibiting tolerogenic pathways or (4) chemically stimulate immunogenic cell death. **(C)** Boosting immunogenic effects. Immunogenicity can be boosted *via* (1) the introduction of cytokines directly or *via* the delivery with (2) oncolytic viruses or (3) CAR T cells. **(D)** Regulating tolerogenic stimuli. Tolerogenic stimuli can be inhibited with (1) antibodies, (2) immune suppressive cells can be forced into differentiation or (3) T cells can be engineered to become resistant to tolerogenic stimuli. This figure was created by authors using Biorender tools (biorender.com).

For example, oxaliplatin induces a more potent immunogenic TCD by the upregulation of extracellular CRT relative to cisplatin (123), which resulted in a significantly higher progression-free and overall survival in patients with gastric cancer (124). These results display that chemotherapeutic agents with similar chemical structures and acting mechanisms can have different outcomes and highlights the possibility to switch from a tolerogenic to immunogenic chemotherapy (**Figure 2A.1**). Hence, understanding the immunogenic properties of cytotoxic agents is necessary to improve the efficacy of combinatory treatments, especially for immunotherapy.

Similar to the wide variety of chemotherapy used, radiotherapy is given in different doses, regimes, and radiation types (125). These parameters influence the immune-activating capabilities of the dying cells. For example, a close association between the dose of radiation and the upregulation of DAMPs has been observed in breast cancer cells (126). This is substantiated with increased survival in patients treated with a higher dose of radiation in small cell lung cancer patients (127). Furthermore, multiple rounds of moderate-dose radiation induce a more potent immune response relative to a single round with a similar total amount of Gray (128). This implies that the optimal radiation dose and fractionation regime are crucial to increase the immunogenic effects of radiotherapy (**Figure 2A.2**). Together, optimization of types and methods used in conventional treatments to increase the immunogenic effect is feasible. This has been shown to increase the survival of cancer patients, presumable by the increase of infiltrating immune cells due to reduced immunosuppression in TME (129). Yet, more research is necessary to determine the optimal effects for specific cancer types and particular genetic alterations.

### Converting Tolerogenic TCD to Immunogenic TCD

**Figure 3:**
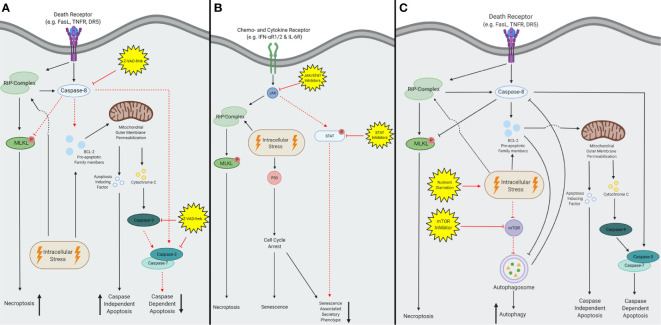
A schematic overview of inhibitors converting tolerogenic cell death to immunogenic cell death. **(A)** The pan-caspase inhibitor Z-VAD-fmk inhibits all caspase activity, the subsequent effects are depicted with red dotted lines. Z-VAD-fmk forces extrinsic stimuli to activate the RIP-kinase complex and thereby initiates necroptosis instead of caspase-dependent apoptosis. Intrinsic stimuli remain able to activate the pro-apoptotic BCL-2 family members and subsequently facilitate mitochondrial outer membrane permeabilization (MOMP). Consequently, both apoptosis inducing factors (AIF) and cytochrome C are released, but only AIF is able to induce apoptosis. Therefore, caspase-dependent apoptosis will be inhibited and decreased, and caspase-independent apoptosis will increase. **(B)** Both Janus kinase- signal transducer and activator of transcription (JAK-STAT) and JAK inhibitors are capable of inhibiting the senescent pathway, depicted in red dotted lines. This reduces the activation of the senescent associated secretory phenotype (SASP) after activation of the extrinsic pathway. In contrast, the intrinsic pathway remains able to induce the SASP *via* activation of p53. **(C)** Both nutrient starvation and mammalian target of rapamycin (mTOR) inhibition increase autophagy, by upregulation of the autophagosome. This figure was created by authors using Biorender tools (biorender.com).

Shifting TCD from a tolerogenic type to an immunogenic type of TCD could be an approach to increase the anti-tumor immune responses. One proposed method is to chemically shift from cell death pathway, by blocking the conventional pathway (**Figure 2B.1**). For example, necroptosis and caspase-independent apoptosis are induced after combining an apoptosis-inducing agent along with caspase-inhibiting drugs (**Figure 3A**) (7, 130). In murine models, TNF-a has been found to induce necroptosis after the incubation with the pan-caspase inhibitor Z-VAD-fmk (131). Furthermore, combining caspase inhibitors with chemotherapy leads to an increase in extracellular DAMPs (132) and results in a decrease of intertumoral T_reg_ cells and an increase in IFN-y secreting cytotoxic T cells.

Additionally, caspase inhibitors are also used along with radiation therapy, which increases TCD (133), but also results in the upregulation of PD-L1 and tumor relapse (134). This effect was effectively controlled by combining radiotherapy, caspase inhibitors and anti-PD-L1. Others have found that the combination of mammalian target of rapamycin (mTOR) inhibitors with radiotherapy and caspase inhibitors results in a potent anti-tumor effect due to an increase of autophagic TCD (135). Additionally, apoptosis induced by IFN-β is converted to necroptosis when either RIP1 or the caspase pathway was inhibited (136). These studies display the possibility to shift from tolerogenic TCD to immunogenic TCD, but clinical efficacy has yet to be proven.

As mentioned above, viruses contain genes that are capable of interfering with cell death pathways. For example, AVs contain a set of genes that are capable of regulating TCD by the sequestering of the pro-apoptotic BCL-2 family (137) and p53 (138), or downregulation of death receptors (139). Moreover, HSVs express proteins that interact with the formation of the autophagosome (140) and inhibit apoptosis (141), some of these genes are deleted during treatment design, to ensure rapid killing of cancer cells (142). These properties can be exploited to convert the mechanisms of cell lysis by oncolytic viruses (**Figure 2B.2**). For example, the upregulation of ﻿E3-11.6K instead of the deletion of ﻿E1B-19K in AVs demonstrates comparable cytotoxicity, but the pathway of cell death activation shifts from caspase-dependent to caspase-independent (143). This shows the possibility of changing cell death pathways directly, which can potentially be used in combination with other cell death-inducing agents. For instance, vaccinia viruses (VVs) express proteins that inhibit caspases directly (144), which can potentially be exploited in a similar manner as the caspase inhibitor Z-VAD-fmk. Furthermore, induction of specific cell death pathways *via* modifications in the autologous genetic composition of the virus saves the limited genomic space and thus provides a possible strategy to convert tolerogenic to immunogenic TCD.

Other strategies have been proposed to target cells with the SASP, where the transformation to this phenotype is blocked (**Figure 2B.3**) (145). For example, inhibition of the Janus kinase- signal transducer and activator of transcription (JAK-STAT) -pathways decreases the number of SASP cells after radiotherapy and chemotherapy in mice (17, 73). These combinatory treatments reduce the levels of tolerogenic chemokines and cytokines, and these positive results lead to the enrolment of a phase II clinical trial combining paclitaxel and ruxolitinib (146). Thus, the transformation of cells to the SASP can be blocked which results in a less tolerogenic TME (**Figure 3B**). Current research will prove whether this enhances the immune infiltration in human tumors and yields beneficial clinical results.

Moreover, the inhibition of autophagy is beneficial as it prevents tumor growth in mice (147) and the tumor cells are more susceptible to radiation therapy (148) as well as various chemotherapies such as cisplatin (149) and temozolomide (150). However, most of these studies were performed using immunodeficient mice and studies in immunocompetent models displayed little effect (151). These discrepancies led to the development of a novel strategy where autophagy is enhanced to increase the immunogenicity of the TME (**Figure 2B.4**) (152–154). Various agents are currently used that mimic effects of fastening in combination with chemotherapy, which has been found to enhance T cell infiltration and depletes T_reg_ cells (155). These studies also demonstrate a significant increase of survival in mice relative to treatment with chemotherapy alone, which resulted in multiple clinical trials (156, 157). Future studies will eventually elucidate the underlying molecular association between autophagy, treatment, and the subsequent immune response (**Figure 3C**).

Taken together, these studies exhibit the possibilities to convert tolerogenic TCD towards immunogenic TCD with the use of specific pathway inhibitors or oncolytic viruses. Some of these inhibitors have already been used in clinical trials for other diseases and have been proven to be safe (158, 159). Thus, current and future research in combining these converting strategies with conventional treatments will provide data about the efficacy of this rationale. If the balance in the TME is found to shift towards an immunogenic environment, new combinations with immunotherapy can be utilized.

### Boosting Immunogenic Effects

The third strategy is boosting the immunogenic effects of treatments using (neo)adjuvant therapies (160). In the past decade, various therapies have been combined with immune checkpoint inhibitors and are effective in some types of cancer (161, 162). This emphasizes the potential to block immune suppressive effects and paved the way for novel strategies that boost immunogenic effects. For example, enhanced immunogenicity can be achieved by the addition of immune stimulatory chemokines and cytokines (**Figure 2C.1**) (163) such as IL-12, which has been shown to increase the overall survival in mammary tumor-bearing mice (164). When IL-12 is administered in combination with an anti-PD-L1 Ab, a synergistic effect is observed. Moreover, chemokines such as GM-CSF are also used along with monoclonal antibodies (mAbs) to boost the immune system, which is shown to increase the overall survival in cancer patients relative to solely mAbs treatment (165, 166). Although initial pre-clinical results often look promising, admission of these immunomodulatory molecules can have a lot of side effects in cancer patients (167). A potential strategy to overcome these side effects could be local administration or delivery of these immunostimulatory molecules (168, 169). Further development of these delivery methods could lead to novel approaches, which allow local modulation of the TME and thereby enhance the clinical results of therapies boosting the immunogenic effects.

One frequently applied method is the delivery of genes expressing immunostimulatory agents with oncolytic viruses (**Figure 2C.2**). This appears to enhance the immune infiltration after the immunogenic lysis by viruses. For example, patients treated with AVs loaded with a gene encoding for GM-CSF show an increase of T cell trafficking to the tumor and induction of T cell-mediated immunity *via* DC cross-presentation (170). Various clinical studies confirmed these results, which led to a successful phase III clinical trial and FDA approval of this therapy (107). Only a minor increase in clinical efficacy was demonstrated, which was suggested to be caused by the upregulation of PD-L1 after treatment with oncolytic viruses (171). These undesired effects are indeed found to be counteracted when this oncolytic virus is combined with an α-PD-L1 antibody since a phase I clinical trial demonstrated improved tumor infiltration of T cells (172). Similar strategies with viruses expressing IL-12 either alone or in combination with other immune modulatory agents have been found to activate NK and T cells and prolong the survival of mice (173, 174). Consequently, a phase I clinical trial is currently being conducted with HSVs expressing IL-12 (175), which will ensure the safety of local IL-12 administration. Together, these findings highlight the possibility to modulate the TME locally with oncolytic viruses together with effective tumor cell lysis.

Comparable strategies are also exploited in CAR T cells, which are genetically modified to secrete immunoenhancing agents (**Figure 2C.3**). For example, CAR T cells secreting IL-12 have shown enhanced anti-tumor efficacy relative to non-secreting CAR T cells, due to increased immune infiltration and activation (176, 177). These promising results led to the enrolment of a phase I clinical trial in patients with ovarian cancer (178, 179). If these clinical studies prove effective immune modulation of the TME, the road will be paved for novel studies testing the efficacy of other immunomodulatory chemo- and cytokines.

In summary, boosting the immunogenicity of treatments was shown to be effective and can result in potent anti-tumor responses. Strategies using immunotherapy with immune-boosting capacities hold great potential for future treatments. Alternative delivery methods of chemo- and cytokines are being developed and will broaden the spectrum of applicable treatments and possible combinations.

### Regulating Tolerogenic Stimuli

Regulating the anticipated tolerogenic stimuli could also be a promising strategy to decrease the immune suppressive effect in the TME. Two major components can be identified as targets which are soluble chemokines and cytokines, or suppressive immune cells. Inhibition of chemo- and cytokines can be achieved by blocking their receptors with inhibitory drugs or antibodies (**Figure 2D.1**).

For example, the CXCL12/CXCR4 axis inhibits the infiltration of cytotoxic T cells, increases the level of T_reg_ cells and promotes metastasis (180). High levels of CXCL12 are secreted by SASP cells, which results in immunosuppression in the TME (181). Blocking these signaling pathways increases the levels of infiltrating cytotoxic T cells and increased survival in mice when combined with immunotherapy (182). Recently, promising results were obtained from a phase I clinical trial in breast cancer patients, where a CXCR4 antagonist was combined with chemotherapy (183). These results provide new rationales to combine these treatments with immunotherapy.

Furthermore, elevated levels of IL-6 have been linked to tumors containing SASP cells (184) and the regulation of IL-6 level has been shown to affect the anti-tumor immunity (185). Multiple studies demonstrated the possibility to interfere IL-6-mediated signaling pathways by either neutralizing the cytokine or blocking the receptor with monoclonal antibodies (186–188). These antibodies are safe for use in human patients (189) and decrease the infiltration of MDSCs in murine solid tumors (190). Currently, multiple clinical trials are examining the efficacy of IL-6 blockade in combination with conventional treatments in cancer patients (191, 192).

Other forms of TCD have also been reported to recruit the immune-suppressive MDSCs (72), and limiting their numbers has been shown to improve the overall survival in patients (193). Another strategy to decrease the tolerogenic effects induced by MDSCs is by differentiating these cells into less immune suppressive cells (**Figure 2D.2**). For example, the delivery of all-trans retinoic acid (ATRA) forces MDSCs to differentiate into dendritic cells or macrophages (194). This results in a significant decrease in circulating MDSCs and consequently enhances the immune response in cancer patients (195). Delivery of MDSC differentiating agents together with conventional immunotherapies is suggested to enhance the anti-tumor immune response and is currently clinically investigated (196).

Moreover, radiotherapy-induced SASP cells are found to attract immunosuppressive effects of MDSCs, which resulted in radiotherapy resistance and decreased T cell efficacy (197, 198). This was suggested to be partially regulated *via* the secretion of TGF-b (199). Two strategies have been found to counteract tolerogenic stimuli with immunotherapy (**Figure 2D.3**), namely T cell transduced with a dormant TGF-b receptor and the use of a specific subset of T cells resistant to TGF-b mediated apoptosis (200, 201). Both strategies demonstrated increased T cell infiltration and effective tumor cell eradication in high TGF-b environments in both mice and humans, which highlights the possibility to produce T cells resistant to tolerogenic stimuli. Thus, selective depletion of suppressive immune stimuli and/or cells is suggested to be feasible and safe. These treatments could be further developed to increase the immunogenic effects in the TME.

Together, these studies underly the possibility to regulate and dampen the tolerogenic effects induced by TCD. Clinical studies that are currently being conducted will show whether these strategies are effective in humans and will provide more knowledge of the tolerogenic effects in the TME.

## Conclusions and Perspectives

Although the mechanisms governing immunogenic TCD are extremely complicated and vary greatly between patients and cancer types, we sought to provide a broad and comprehensive overview. Notably, caspase-independent apoptosis and necroptosis are the most prevalent forms of immunogenic TCD and can direct the balance of the TME towards an immunostimulatory and activating environment. These forms of TCD are therefore suggested to be more beneficial in terms of anti-tumor immunity, which presents an appealing rationale to combine conventional therapies with strategies that promote these types of TCD. Despite the astonishing steps forward in the field of combinatory treatments with immunotherapy, we still have a long way ahead of us before we can achieve effective treatments for all types of cancer. We sought to present four substantiated methods to tailor therapeutics more effectively based on the current knowledge; avoiding tolerogenic TCD, converting tolerogenic TCD to immunogenic TCD, boosting the immunogenic effects and regulating tolerogenic effects. Some of these strategies have been proven to be effective and safe in patients and others are presently under clinical investigation. Whereas current combinatory therapies already provide substantial benefits in patients, combining one or more of these four strategies with immunotherapy will ensure a foundation for a novel era of treatments.

The recent advances in gene editing techniques open up a new discipline of research and allow us to modulate the TME more easily and precisely through sophisticated delivery. Although the delivery of immunomodulatory agents with CAR T cells or oncolytic viruses is in its infancy, these strategies have the potential to provide vigorous methods to disrupt the TME together with the induction of TCD. Increasing knowledge enables opportunities to expand the immunomodulatory possibilities of cell-based therapies. For example, inducible CAR T cell delivery systems have been proven to be robust (177) and might be employed to exploit time-dependent effects of conventional treatments or modulate the TME over time. Furthermore, cellular delivery systems are currently designed to become resistant to the suppressive TME (200) and are also used to deliver (biological) cytotoxic substances with immunomodulatory cells (202). Such immunological-based therapies have yet to be proven to be effective in cancer patients but hold great potential for the future of cancer treatment.

A major hurdle in the pursuit of novel therapy design based on therapy-induced TCD is the inter-patient heterogeneity because consequences of treatment on the TME are patient-specific. For instance, the genetic composition of tumor cells has a considerable impact on the signaling cascade governing TCD and defects in these pathways have been observed frequently (203, 204). These genetic alterations can potentially be used as biomarkers for the design of patient-specific combinatory therapies (205). Besides predictive biomarkers, recent studies have hypothesized the correlation between the immunogenicity of therapy and the level of circulating HMGB1 in various tumor types (206, 207). This method holds the potential to measure the impact of a certain therapy in patients in real-time, but more evidence is needed to uncover the relationship between circulating biomarkers and types of cell death. Development of more accurate methods to assess immunogenicity in patients could also shed a light on the effects of other patient-specific facets such as mutational burden and tumor site, which are thought to also play a huge role in the anti-tumor immune response (208, 209). Moreover, other varying parameters within the tumor such as acidity, hypoxia and nutrient deprivation are also thought to affect pathways of TCD and release of DAMPs (210–212).

Similarly, the cellular composition of the tumor stroma plays a key role in treatment efficacy (213) and should therefore not be overlooked. A growing interest in stroma cells has led to the development of treatments targeting neovascular and fibroblasts (214, 215). This top-down approach focuses on perturbation of the TME instead of designing therapies to overcome environmental challenges. These efforts might lead to resources that allow uniformization of inter-patient TMEs pre-treatment by disrupting certain suppressive structures and enable the use of universal therapies.

For the sake of clarity, this review has made a clear distinction between tolerogenic and immunogenic effects of TCD, but it should be noted that these are two extremes on a wide spectrum. A vast number of interactions determine the balance in the tumor microenvironment and therefore, generalization of immunoregulatory concepts continues to be difficult.

Contributing to this challenge is the focus on a small subset of DAMPs, namely ATP, HMGB1 and CRT. Although it is widely recognized that currently known immunogenic and tolerogenic molecules are only a fraction of actual regulatory signals, progression in the field is slow. Garg and Agostinis (216) summarized all known DAMPs currently known, but the effects of most of these molecules remain understudied. The effects of DAMP release and immunogenic TCD on maturation and activation of immune cells in distant organs such as the lymph nodes and the bone marrow remain yet to be studied. Although it has been shown that inhibiting the release of tolerogenic signals after chemotherapy-induced TCD induces vast changes in the tumor draining lymph nodes, little is known about the relationship between therapy-induced TCD, systemic immune alterations and therapeutical outcome (217, 218). Novel research strategies can change the perception of the immunogenic and tolerogenic effects of TCD and can result in clarification of poorly understood mechanisms and unaccountable results. Furthermore, the relationship of DAMPs with non-immune stromal cells and tumor cells should be investigated more thoroughly, because some studies suggest extensive crosstalk mediated by these molecules (219, 220). This again underscores the relatively unknown effects of anti-cancer therapy on the non-malignant stromal cells but highlights the overwhelming complexity of interactions in the TME.

Furthermore, a clearer picture of the effects of certain types of TCD and immunomodulatory agents on the TME is necessary to increase our knowledge of this complicated system. Multiple studies have shown opposing results, which could be addressed with novel high-throughput techniques such as single cell RNA-seq (221). More thorough discrimination between forms of cell death has resulted in a broader spectrum of known cell death pathways, which can be implemented in future research (222). Moreover, adequate immune-competent models will shine a light on conventional and novel treatments and will pave the way for quicker translation to the clinic. Especially the gap of knowledge in the field of molecular radiotherapy holds potential for improvement. With our growing understanding of biomolecular mechanisms, clinical research is obliged to focus on the aspects underlying treatment efficacy. An increasing number of clinical trials are enrolled with combinatory immunomodulatory therapies, which could provide tremendous amounts of valuable information if properly examined. Therefore, the collaboration between fundamental, translational and clinical sciences is fundamental for comprehension of this ever-growing field of biomedical research. In the end, cancer therapies will only be fully successful if the mechanisms governing TCD and immune interactions in the TME are fully understood.

## Author Contributions

TS, K-SC and KS researched data for the article. All authors contributed to writing the article the article and approved the submitted version.

## Funding

This work was supported by NIH grants R01-NS121096 (KS) and R01-NS107857 (KS).

## Conflict of Interest

KS owns equity in and is a member of the Board of Directors of AMASA Therapeutics, a company developing stem cell based therapies for cancer. KS’s interests were reviewed and are managed by Brigham and Women’s Hospital and Partners HealthCare in accordance with their conflict of interest policies.

The remaining authors declare that the research was conducted in the absence of any commercial or financial relationships that could be construed as a potential conflict of interest.
